# Degree difference: a simple measure to characterize structural heterogeneity in complex networks

**DOI:** 10.1038/s41598-020-78336-9

**Published:** 2020-12-07

**Authors:** Amirhossein Farzam, Areejit Samal, Jürgen Jost

**Affiliations:** 1 Max Planck Institute for Mathematics in the Sciences, 04103 Leipzig, Germany; 2 The Institute of Mathematical Sciences (IMSc), Homi Bhabha National Institute (HBNI), Chennai, 600113 India; 3 The Santa Fe Institute, Santa Fe, NM 87501 USA

**Keywords:** Complex networks, Statistical physics

## Abstract

Despite the growing interest in characterizing the local geometry leading to the global topology of networks, our understanding of the local structure of complex networks, especially real-world networks, is still incomplete. Here, we analyze a simple, elegant yet underexplored measure, ‘degree difference’ (DD) between vertices of an edge, to understand the local network geometry. We describe the connection between DD and global assortativity of the network from both formal and conceptual perspective, and show that DD can reveal structural properties that are not obtained from other such measures in network science. Typically, edges with different DD play different structural roles and the DD distribution is an important network signature. Notably, DD is the basic unit of assortativity. We provide an explanation as to why DD can characterize structural heterogeneity in mixing patterns unlike global assortativity and local node assortativity. By analyzing synthetic and real networks, we show that DD distribution can be used to distinguish between different types of networks including those networks that cannot be easily distinguished using degree sequence and global assortativity. Moreover, we show DD to be an indicator for topological robustness of scale-free networks. Overall, DD is a local measure that is simple to define, easy to evaluate, and that reveals structural properties of networks not readily seen from other measures.

## Introduction

Since the dawn of network science^1–3^, scientists have tried to capture the structure and dynamics of networks by measures that are simple to understand and easy to evaluate (see e.g.^4–6^). Early studies on the structure of complex networks focused primarily on the global topology of these discrete objects^7–9^. Global measures necessarily take some kind of average, and therefore, such measures do not capture much of the individual variability and heterogeneity in networks. To avoid this, it is important to investigate local measures and their distributions in complex networks. Local measures are in particular of substantial interest for analyzing massive networks, where global measures are either impractical to compute or fail to provide the desired information about network components. It is thus natural to witness growing interest among the network science community to investigate the local geometry of complex networks (see e.g.^10–14^). Local clustering coefficient^7^, generalized degree, local assortativity^15^, Ollivier–Ricci curvature^11,12,14,16^, and Forman–Ricci curvature^13,14^ are some of the notable measures characterizing the local structural properties of complex networks.

Moreover, with the recent exception of discrete Ricci type curvature measures, such local or global measures are typically evaluated on vertices, rather than on edges, although the edges are of course what really constitutes a network. In this work, we shall therefore systematically pursue an edge-based approach to characterize the local structure of complex networks. As mentioned, discrete Ricci type curvature measures are local and edge-based, and they are by now established as useful tools for the analysis of empirical networks^12–14,17,18^. For instance, the Forman-Ricci curvature of an edge in an unweighted and undirected network essentially evaluates the sum of the degrees of its two vertices, and edges with a large such sum are important for the cohesion of the network in question and therefore deserve attention. However, when we want to understand the local heterogeneity in a network, Forman–Ricci curvature may not be so useful, because it does not distinguish between an edge that connects two vertices of intermediate but similar degrees, from an edge that connects a highly connected vertex with a sparsely connected one; in both cases, the sum of their degrees is large. Now, there is an important and well-established global concept for judging the homogeneity or heterogeneity of a network, its *assortativity* (see for instance^19^). A network is assortative if on average, the degrees of connected vertices are similar, and disassortative, if they tend to be rather different. For instance, many social networks, particularly those formed through group-to-group connections, are known to be assortative^20^, i.e. agents with high degree seem to connect to other high-degree agents, and similarly, low-degree agents tend to connect to agents with lower rather than higher degree. Again, this property cannot be captured by node-based quantities, such as the degree sequence, because a simple rewiring can transform an assortative into a disassortative network or vice versa, without changing the degree sequence. This motivates us to systematically explore the ‘degree difference’ between two vertices of an edge in complex networks.

Degree difference or closely related measures have been previously used in the study of complex networks, for instance, to study scale-free properties of networks^21^, and to investigate the structure of inter-organizational networks^22^. However, curiously, so far there seems to have been no systematic analysis of degree difference in complex networks, although for the reasons explained above, this is a most natural local measure. It is simple to define, easy to evaluate, and captures the local picture underlying assortativity or disassortativity. Moreover, we shall show in this contribution that the measure provides novel insight into both synthetic and real networks. For the synthetic networks, we will derive explicit formulae, thereby laying the foundations for a theoretical investigation.

In fact, assortativity can be defined more generally^23^ to express how similar or dissimilar neighbouring vertices are with respect to some quantity $\alpha$. In particular for social networks^19^, this is important as it connects to homophily, that is, the tendency to associate with like-minded or otherwise similar people. Thus, for a graph *G*(*V*, *E*), with vertex set *V* and edge set *E*, and an attribute $\alpha : V \rightarrow {} {\mathbf {F}}$, mapping the *n* vertices in *V* to elements in ${\mathbf {F}}$, assortativity captures heterogeneity in mixing patterns in *G* at a global scale. The global assortativity (GA) with respect to $\alpha$ is given by

1 $$\begin{aligned} r_{\alpha } = \frac{Tr(e) - \Vert {e^2}\Vert _{L_1}}{1 - \Vert {e^2}\Vert _{L_1}} \end{aligned}$$

where *e* is the $n \times n$ matrix of joint probabilities with $e_{i,j} = P(\alpha (i), \alpha (j))$, *Tr*(*e*) is the trace, and $\Vert {e^2}\Vert _{L_1}$ is the $L_1$ norm of *e*. When $\alpha$ maps the vertices to their degrees, we denote $r_{\alpha }$ by *r*, and Eq. (1) is equal to the Pearson correlation coefficient of the degrees of connected vertices. By convention, if the term *assortativity* is used without specifying the attribute, the attribute is assumed to be the degree.

There have been previous attempts to break assortativity down to its more local components. Piraveenan et al.^24^ define a local point-wise measure of assortativity, local node assortativity (LNA), denoted by $\hat{\rho }_v$, quantifying the contribution of each vertex *v* to the GA in the network as follows

2 $$\begin{aligned} \hat{\rho }_v = \frac{j(j+1)(\bar{k}_v-\mu _q)}{2M{\sigma _q}^2} \end{aligned}$$

where *j* is the excess degree of *v*, $\bar{k}_v$ is the average excess degrees of the neighbours of *v*, $\mu _q$ is the global average excess degree, *M* is the number of edges in the network, and $\sigma _q$ is the standard deviation of the excess degree distribution in the network. Consider an edge $e=\{v, u\}$. As we arrive at *v* via the edge *e*, excess degree of *v*, also referred to as *remaining degree* of *v*, is the number of neighbours of *v* other than the vertex *u* from which we arrived at *v*. Given how excess degree is defined, it is the natural measure to consider when comparing the number of neighbours of two connected vertices, since the connection between the two is a given and the connections other than the edge between the two neighbours are those contributing to the similarity or difference in their degrees. Hence, excess degree of the vertices is the measure used for computing GA and LNA. Note that, although conceptually excess degree of a vertex is defined with an incident edge in mind, formally excess degree is, in fact, equal to degree minus 1.

GA can be obtained from LNA through the following identity

3 $$\begin{aligned} r&= \sum _{j, k \in D(V)}\frac{jk(e_{j,k} - q_j q_k)}{\sigma _q ^2} \nonumber \\&= \frac{1}{\sigma _q ^2} \left[ \left( \sum _{j,k \in D(V)} jk e_{j,k} \right) - \mu _q ^2 \right] \end{aligned}$$

4 $$\begin{aligned}&= \frac{1}{\sigma _q ^2} \ \sum _{j} \sum _{v \in V_j} \left( \left[ (j+1) \frac{j \bar{k}_v}{2M} \right] - \left[ (j+1) \frac{j\mu _q}{2M} \right] \right) \nonumber \\&= \frac{1}{\sigma _q ^2} \ \sum _{j} \sum _{v \in V_j} \hat{\rho }_v \end{aligned}$$

where *D*(*V*) denotes the set of degrees of vertices in *V*, and $V_j$ is the set of vertices in *V* with excess degree *j*. In Eq. (4), the term within the first braces is the contribution of vertex *v* to the first term in Eq. (3) and the term within the second braces is its contribution to $\mu _q ^2$.

**Figure 1:**
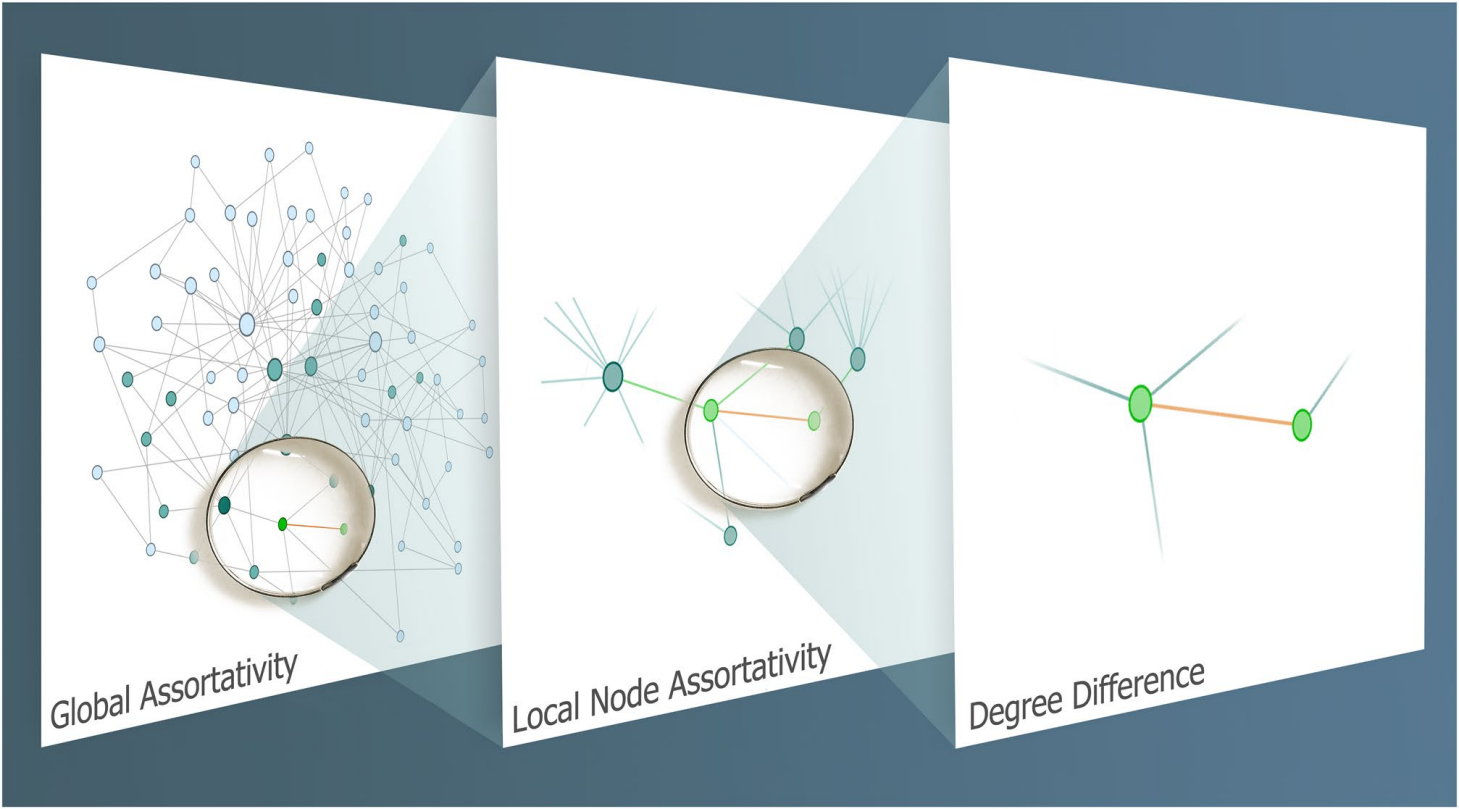
Heterogeneity in mixing patterns in networks is quantified through global assortativity (GA) given by Eq. (1) at the global scale. At a more local scale, mixing pattern is quantified through local node assortativity (LNA) given by Eq. (2) which aggregates the heterogeneity in degrees of the vertices anchoring the edges incident on the vertex. Degree Difference (DD) is the basic local unit contributing to assortativity and captures heterogeneity in mixing patterns at the scale of individual edges.

**Figure 2:**
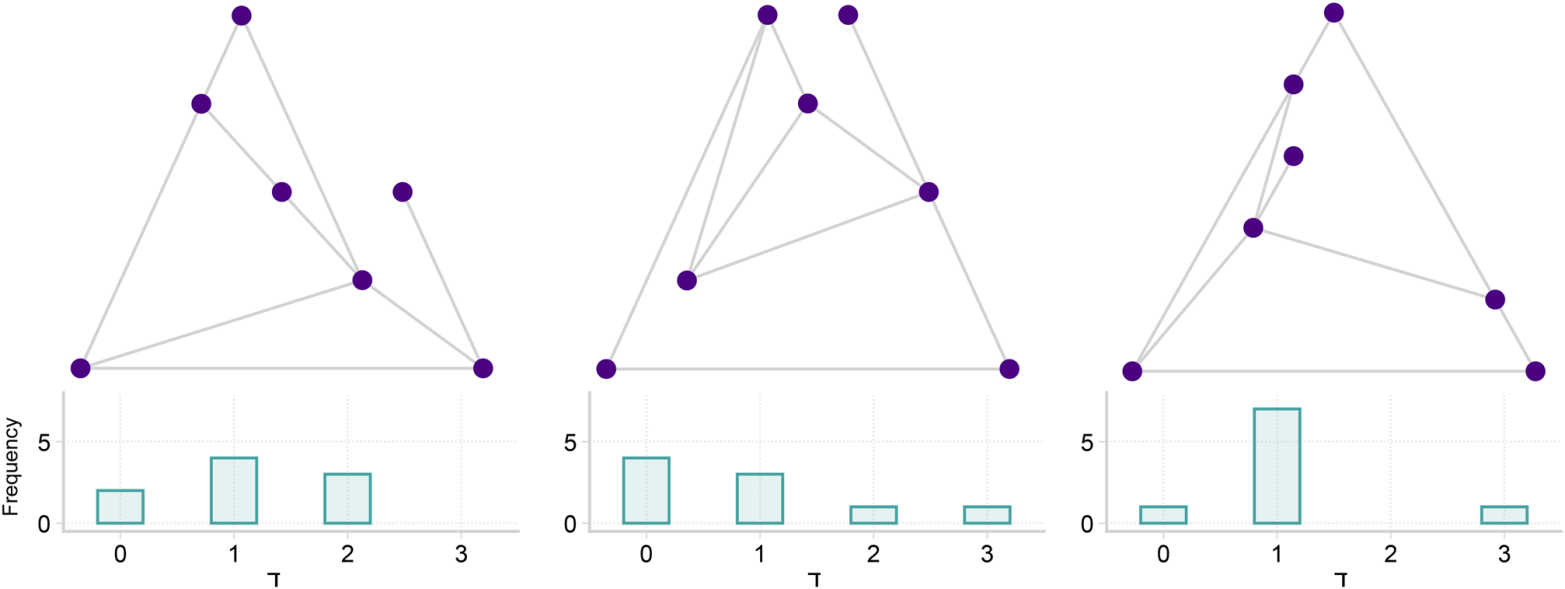
Graphs with same degree sequence and same global assortativity (GA) can have different degree difference (DD) distributions. The figure shows 3 graphs with 7 vertices and 9 edges with same degree sequence and GA of $\approx -0.358$, but with different DD distributions. In the figure, the bar plot below each graph shows the DD distribution.

While this represents a valuable step towards understanding local mixing patterns in networks, LNA appears somewhat complicated and is defined on the vertices. In fact, at first sight it seems most natural to come up with such a node-based measure. But recall that assortativity is evaluating similarities or differences between neighbouring vertices, and two neighbouring vertices are nothing but an edge. Therefore, it seems more natural to evaluate quantities directly on edges. That is, in fact, our starting point. Thus, we shall decompose assortativity into its basic unit, the degree difference (DD) between the vertices forming an edge. Given an edge $e=\{v,u\}$ in an unweighted and undirected graph linking the vertices *v* and *u* with degrees ${\text {deg}}(v)$ and ${\text {deg}}(u)$, DD of *e* is given by

5 $$\begin{aligned} \daleth (e)&= \left| {\text {deg}}(v) - {\text {deg}}(u) \right| \end{aligned}$$

where $\daleth : E \rightarrow {} {\mathbb {Z}}^{\ge }$ is a function from the edge set of the graph to non-negative integers, mapping *e* to absolute value of its DD. Similarly, for directed graphs, we define directed DD (diDD) as follows

6 $$\begin{aligned} \daleth _{\rightarrow {}}(e)&= {\text {deg}}^{out}(u) - {\text {deg}}^{in}(v) \end{aligned}$$

where $e=(v, u)$ is the directed edge from *v* to *u* and $\daleth _{\rightarrow {}}: E \rightarrow {} {\mathbb {Z}}$ has the entire set of integers as its codomain. Note that there are four possible ways to define the directed DD (diDD), corresponding to the four permutations of in- or out-degree of the head vertex minus in- or out-degree of the tail vertex. The variation given in Eq. (6) is most consistent with the orientation on the edge and the direction of potential flow. After verifying that this simple and elegant network measure meaningfully captures structural similarities and differences, we here show that DD is independently informative and capable of characterizing network structure. Importantly, DD of edges, through (indirect) quantification of the contribution of individual edges to the GA, is the basic unit of assortativity, as illustrated in Fig. 1. Furthermore, we provide an explanation as to why DD can characterize structural heterogeneity in mixing patterns, a feature that is lost due to averaging when employing the measures GA or LNA. In Fig. 2, we show three graphs with same degree sequence and same GA that have different DD distributions.

The remainder of this paper is organized as follows. In the next section, we derive the analytical formulae for DD distribution in Erdös–Rényi (ER) random graphs and Barabási–Albert (BA) scale-free graphs. We also show the connection between DD distribution and GA. Thereafter, in the “Computational results” section, we present our numerical results for the DD distribution in diverse synthetic and real networks. We also report our computations showing the importance of DD for topological robustness in networks. Lastly, we conclude with a summary and future outlook.

## Analytical results

Based on the definition of DD for an edge $e=\{v,u\}$ in an undirected and unweighted network given by Eq. (5), the probability mass function $P_{\daleth }$, where $P_{\daleth }(d) = Pr(\daleth (e) = d)$, is given by

7 $$\begin{aligned} P_{\daleth }(d)&= Pr \left( | {\text {deg}}(v) - {\text {deg}}(u) | = d ~ \left| \right. ~ \{v, u\} \in E \right) \nonumber \\&= \sum _{\begin{array}{c} \{v, u\} \in E \\ s.t. |k-l| = d \end{array}} Pr \left( {\text {deg}}(v)=k ~ , ~ {\text {deg}}(u)=l ~ | ~ \{v, u\} \in E \right) . \end{aligned}$$

We next derive the analytical formulae for the DD distribution in two widely-used network models. These formulae will express DD distribution as a sum for Erdös–Rényi (ER) random graphs^25^ and Barabási–Albert (BA) scale-free networks^8^. Thereafter, we present our analytical calculations unravelling the connection between DD, LNA and GA in undirected networks.

### DD distribution for Erdös–Rényi model

In an ER random graph, *G*(*n*, *p*), where *n* is the number of vertices and *p* is the probability that an edge exists between any pair of vertices, the degrees of two neighbouring vertices are uncorrelated except for the edge that is connecting them. Therefore, the relevant quantity is the *excess degree* of a vertex and we denote its probability distribution by $q_{k} := Pr({\text {exdeg}}(v)=k) = Pr({\text {deg}}(v)=k+1)$. For a pair of vertices *v* and *u* connected via an edge $\{v,u\}$, we then have the identity^26^

8 $$\begin{aligned} Pr({\text {deg}}(v)=k, {\text {deg}}(u)=l ~ | ~ \{v, u\} \in E) ~&= ~ q_{k-1} \ q_{l-1}. \end{aligned}$$

The above relation holds since, conditional on the existence of an edge between two vertices, their excess degree distributions are independent. For given degree and excess degree distributions in *G*(*n*, *p*)^23^, Eqs. (7) and (8) imply

9 $$\begin{aligned} P_{\daleth }(d) ~&= ~ \sum _{|k - l| = d} q_{k-1} \ q_{l-1} \nonumber \\&= ~ \sum _{|k - l| = d} B_{k-1}^{n-2} \ B_{l-1}^{n-2} \ p^{k+l-2} \ {(1-p)}^{2n-2-(k+l)} \nonumber \\&= ~ (2 - \delta _{d, 0}) \ p^{d-2} \ (1-p)^{2(n-1)-d} ~ \sum _{l = 1}^{n-1-d} \ B_{d+l-1}^{n-2} \ B_{l-1}^{n-2} \ \left( \frac{p}{1-p} \right) ^{2l} \end{aligned}$$

where $B^n_k$ denotes the binomial coefficient $$\left( {\begin{array}{c}n\\ k\end{array}}\right) $$ and $\delta _{d, 0}$ is the Kronecker delta, which we use to avoid double counting the same permutation of (*k*, *l*) when $d=0$.

As $n\rightarrow {+\infty }$, the degree distribution for the graph ensemble *G*(*n*, *p*) with average degree $c~=~p(n-1)$ becomes the Poisson distribution^23^

10 $$\begin{aligned} p_k&= e^{-c} \frac{c^k}{k!}. \end{aligned}$$

where $p_k$ is the probability that a given vertex has degree *k*. As $q_k = \frac{(k+1)p_{k+1}}{c}$ for ER random graphs, the excess degree distribution is given by

11 $$\begin{aligned} q_{l-1}&= e^{-c} \frac{c~^{l-1}}{(l-1)!}. \end{aligned}$$

Inserting this in Eq. (9), for sufficiently large ER random graphs, we can approximate DD distribution by

12 $$\begin{aligned} P_{\daleth }(d) ~&= ~ \sum _{|k - l| = d} e^{-2c} \frac{c~^{k-1}}{(k-1)!} \ \frac{c~^{l-1}}{(l-1)!} \nonumber \\&= ~ (2 - \delta _{d, 0}) \ e^{-2c} \ c^{d-2} \ \sum _{l = 1}^{n-1-d} \frac{c^{2l}}{(d+l-1)! \ (l-1)!} . \end{aligned}$$

**Figure 3:**
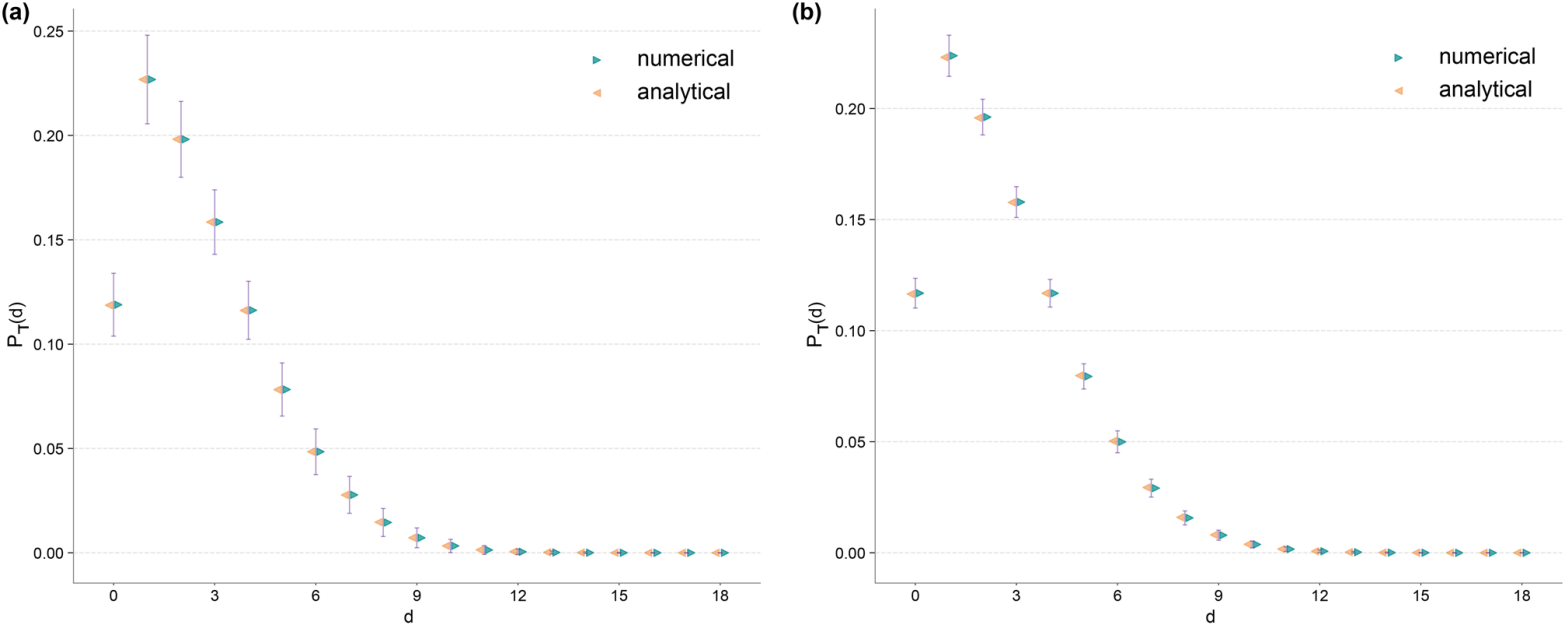
Concordance between analytical formula for DD distribution and numerical computations in Erdős–Rényi (ER) model. (**a**) Computations for ER graph with 200 vertices and average degree $\sim 6$. In this case, Eq. (9) is used to obtain the analytical result. (**b**) Computations for ER graph with 1000 vertices and average degree $\sim 6$. In this case, Eq. (12) is used to obtain the analytical result. In both cases, the numerical result is obtained via averaging the computed $P_{\daleth }$ over an ensemble of 10,000 ER networks with same size and average degree. Note that $P_{\daleth }(d)$ is of the order of $10^{-6}$ or smaller for $d>18$, thus given our ensemble size, the comparison between the numerical and analytical computations is not valid, and thus not shown, for $d>18$.

In Fig. 3, we verify that the formulae given by Eqs. (9) and (12) match with the numerical computations for values of *d* where $P_{\daleth }(d)$ is sufficiently large considering the ensemble size.

### DD distribution for Barabási–Albert model

To derive the DD distribution in a BA network from Eq. (7), we use a result by Fotouhi and Rabbat^27^ for the joint degree distribution of neighbouring vertices in a BA network with $n \rightarrow {} \infty$, and this result is

13 $$\begin{aligned} Pr\left( {\text {deg}}(v)=k, \ {\text {deg}}(u)=l \ | \ \{v, u\} \in E \right) ~&= ~ ~ \frac{2 \ \beta \ (\beta + 1)}{k \ (k+1) \ l \ (l+1)} ~ \left[ 1 ~ - ~ B_{\beta + 1}^{2 \beta + 2} \ \frac{B_{l - \beta }^{k + l - 2\beta }}{\ B_{l+1}^{k+l+2} \ } ~ \right] \end{aligned}$$

where $\beta$ gives the number of edges attached to the new vertex added at each iteration of the BA model implementing a preferential attachment scheme. Thereafter, using Eq. (7), we can obtain the following analytical formula for the DD distribution in BA networks

14 $$\begin{aligned} P_{\daleth }(d) ~&= ~ \sum _{|k - l| = d} \ ~ \frac{2 \ \beta \ (\beta + 1)}{k \ (k+1) \ l \ (l+1)} ~ \left[ 1 ~ - ~ B_{\beta + 1}^{2 \beta + 2} \ \frac{B_{l - \beta }^{k + l - 2\beta }}{\ B_{l+1}^{k+l+2} \ } ~ \right] . \end{aligned}$$

### Connection with global assortativity

The connection between DD and GA is clear once the identity in Eq. (4) is understood. For a graph *G*(*V*, *E*), the following identities explain the connection between GA and DD distribution with LNA as an intermediate step.

15 $$\begin{aligned} \sigma _q ^2 \ r&= \sum _{j} \sum _{v \in V_j} \left( \left[ (j+1) \frac{j \bar{k}_v}{2M} \right] - \left[ (j+1) \frac{j\mu _q}{2M} \right] \right) \end{aligned}$$

16 $$\begin{aligned}&= \sum _{j} \sum _{v \in V_j} \left( \left[ \frac{j(j+1)}{2M} \left( \frac{\sum _{k\ge j}(k-j) - \sum _{k< j}(j-k)}{j+1} + j \right) \right] - \left[ (j+1) \frac{j\mu _q}{2M} \right] \right) \nonumber \\&= \sum _{j} \sum _{v \in V_j} \left( \left[ \frac{j^2 (j+1)}{2M} + \frac{j\sum _{k \ge j}(k-j) - j\sum _{k< j}(j-k)}{2M} \right] - \left[ (j+1) \frac{j\mu _q}{2M} \right] \right) \nonumber \\&= \left[ \sum _{j} \left( N q_j \frac{j^2 (j+1)}{2M} \right) + \left( \sum _{j} \sum _{d} \left[ \sum _{k=j \pm d} \frac{|k-j|}{2M} + \sum _{k=j+d} \frac{(j-1)(k-j)}{2M} - \sum _{k=j-d} \frac{(j+1)(j-k)}{2M} \right] \right) \right] \nonumber \\&~~~~~ - \left[ \sum _j \sum _{v \in V_j} \left( (j+1) \frac{j\mu _q}{2M} \right) \right] \nonumber \\&= \left[ \sum _d d P_{\daleth }(d) + \frac{1}{2M} \sum _{j} \left( N q_j j^2 (j+1) + (j-1) \sum _{k \ge j} (k-j) - (j+1) \sum _{k < j} (j-k) \right) \right] - \left[ \sum _j \sum _{v \in V_j} \left( (j+1) \frac{j\mu _q}{2M} \right) \right] \nonumber \\&= \left[ \langle d\rangle + \frac{N}{2M} \langle j^2 (j+1)\rangle + \frac{1}{2M} \sum _{j} \sum _d \left( \sum _{k=j+d} \left[ d(j-1) \right] - \sum _{k=j-d} \left[ d(j+1) \right] \right) \right] - \left[ \sum _j \sum _{v \in V_j} \left( (j+1) \frac{j\mu _q}{2M} \right) \right] \end{aligned}$$

where $V_j$ denotes the set of vertices with excess degree *j*, *N* is the number of vertices in the network, *d* is the DD, $P_{\daleth }$ is the probability mass function of DD distribution, and the remaining notation is as in Eq. (2). Note, the index *k* under the summations in above equation refers to the excess degree of only the neighbours of the vertex with excess degree *j*, and we used this incomplete notation for brevity. The precise expanded notation, for $k=j+d$ for instance, is $\{ ~ k \in \Xi _v ~~ | ~~ k=j+d ~ \}$, where *v* is a vertex with excess degree *j* and $\Xi _v$ is the set of excess degrees of neighbours of *v*. Also, note that the degree difference is the same as the excess degree difference of two neighbouring vertices. In Eq. (16), the terms in the first outer brackets show the connection between DD and the first term in the summation in Eq. (4), and the term in the second outer brackets is the same as the second term in the summation in Eq. (4), i.e., the contribution of individual vertices to $\mu _q ^2$. The first outer brackets in Eq. (16) contains the first moment of DD distribution (first term in the brackets), the third and second moments of excess degree distribution (the second term in the brackets), and a sum involving DD and excess degree of an incident vertex (the third term in the brackets). In addition to explaining the connection between DD and GA, Eq. (16) further clarifies that we can compute GA and LNA using DD and excess degrees, while DD cannot be deduced from GA and LNA. We demonstrate this remark in supplementary information (SI) Figure S1 where we show DD distribution in an ensemble of BA networks as the network is rewired to increase its GA, and in SI Figure S2 where we set a constraint on GA of ensembles of ER and BA networks and show DD distribution after two random independent rewirings.

**Figure 4:**
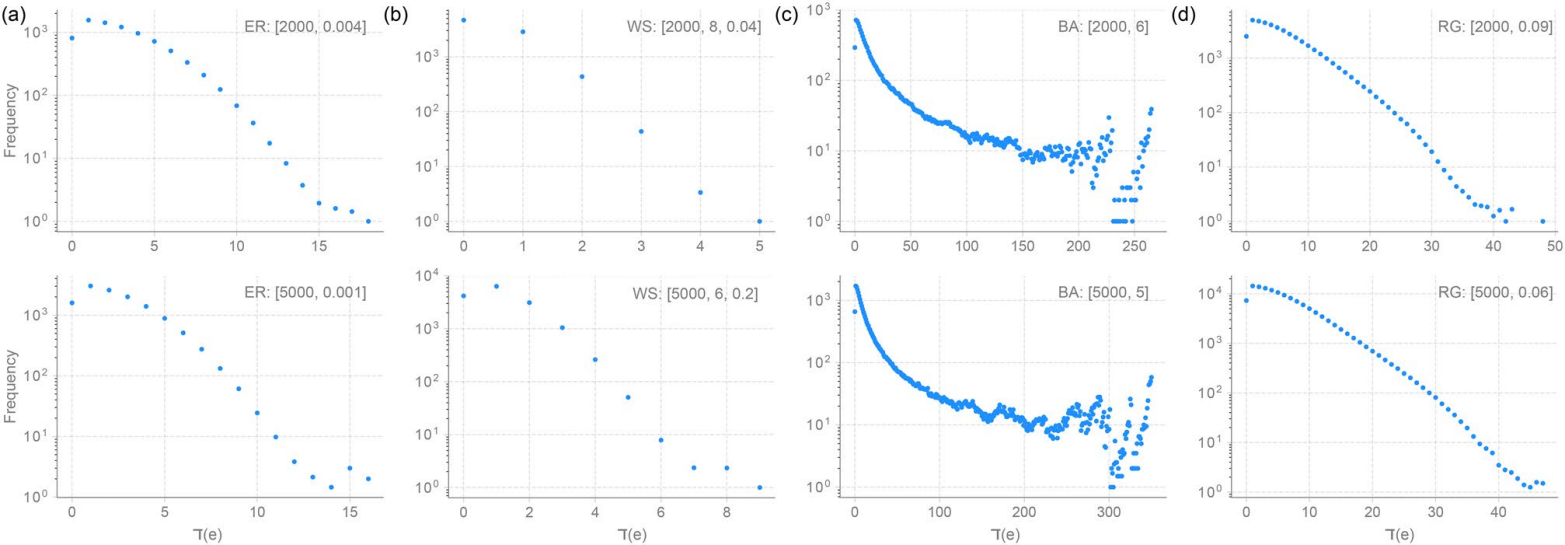
DD distributions in 4 different synthetic networks. (**a**) Erdös–Rényi (ER). (**b**) Watts–Strogratz (WS). (**c**) Barabási–Albert (BA). (**d**) Random Geometric (RG). For each model, the parameters used are indicated besides it in parenthesis. For ER model, the parameters are number of vertices *n* and probability *p* of connecting an edge between any pair of vertices. For WS model, the parameters are number of vertices *n*, the number of neighbours *k* to which each vertex is connected in the starting regular graph, and rewiring probability $\beta$. For BA model, the parameters are number of vertices *n* and number of edges $\beta$ that are attached to the new vertex at each iteration step. For RG model, the parameters are number of vertices *n* and radius $\epsilon$. The reported correlation for each model and a given set of parameters is an average over a sample of 50 networks.

## Computational results

We computed the DD distribution for 4 synthetic networks^7,8,25,28^, namely, Erdös–Rényi (ER) model, Watts–Strogatz (WS) model, Barabási–Albert (BA) model, and Random Geometric (RG) model, and 10 empirical or real networks^7,29–37^. Of the 10 real networks analyzed here, 6 are undirected and 4 are directed networks. The full description of the network dataset is included in the SI Appendix. We also use the 4 synthetic networks to analyze the relationship between DD and topological robustness and to investigate the possible correlation between DD and other edge-based measures.

**Figure 5:**
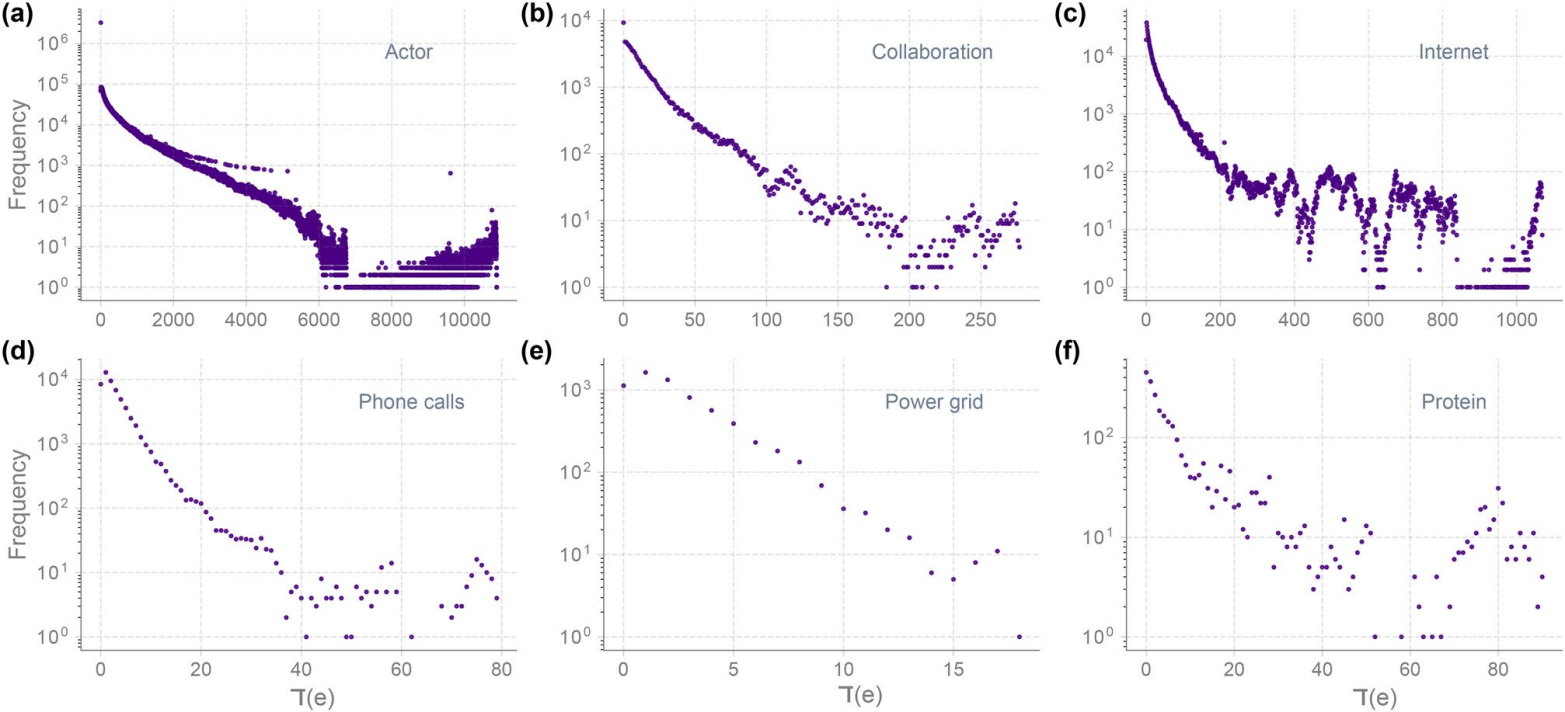
DD distributions in 6 undirected real-world networks. (**a**) Actor. (**b**) Collaboration. (**c**) Internet. (**d**) Phone calls. (**e**) Power grid. (**f**) Protein.

**Figure 6:**
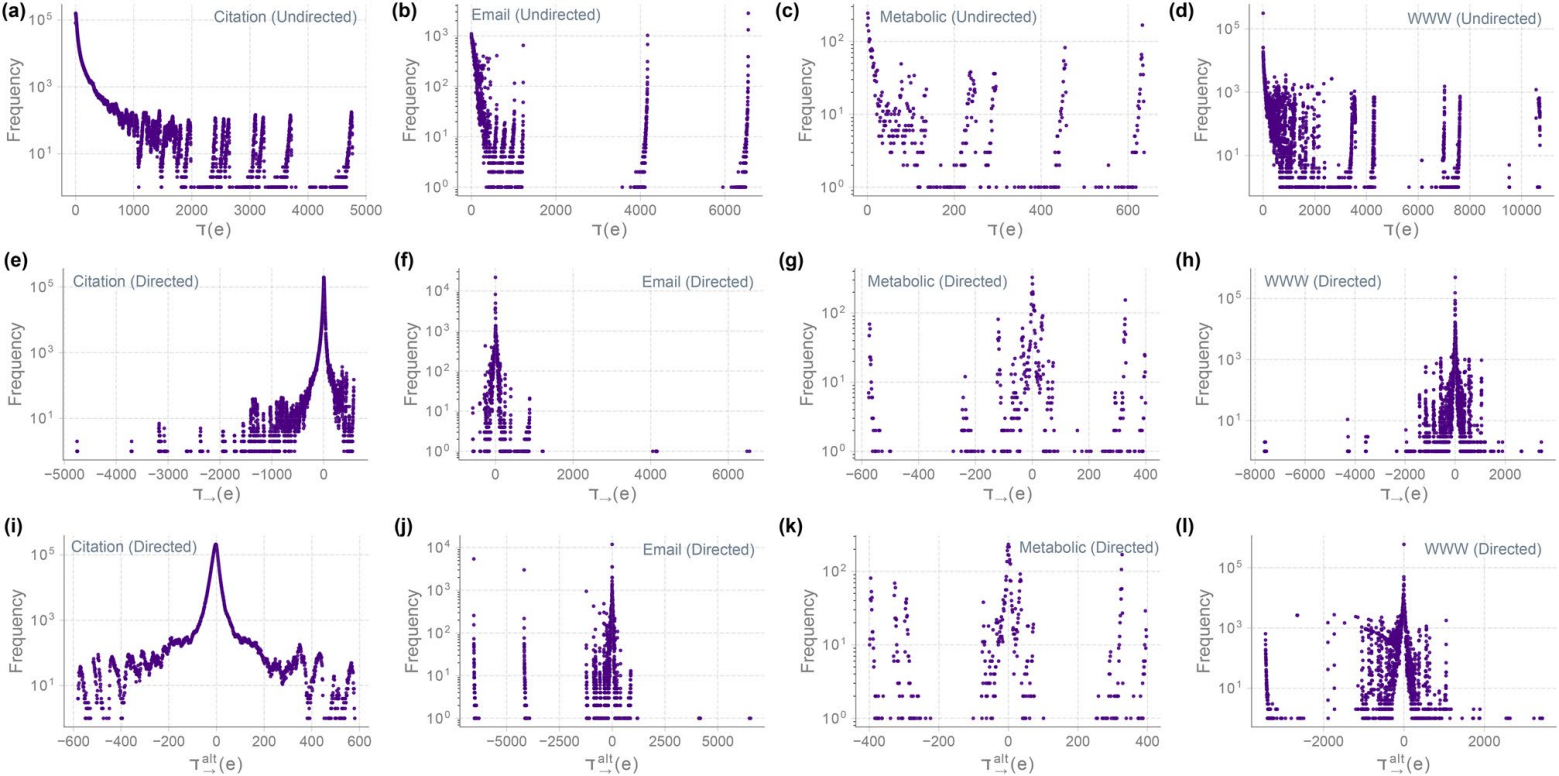
DD distributions in 4 directed real-world networks. (**a**–**d**) Distributions $\daleth (e)$ computed using Eq. (5) by ignoring the directions of the edges. (**e**–**h**) Distributions $\daleth _{\rightarrow {}}(e)$ computed using the directed definition Eq. (6). (**i**–**l**) Distributions $\daleth ^{alt}_{\rightarrow {}}(e)$ computed using an alternative definition of diDD.

### DD distribution in undirected networks

We have computed the DD distribution of edges in 4 undirected synthetic networks and 6 undirected real networks listed in SI Appendix (Figs. 4, 5). From these figures, we can observe qualitative differences between the DD distribution in different undirected networks. As the DD distributions in Fig. 4 suggest, different types of synthetic networks have distinct DD distributions. In particular, random geometric (RG) graphs are known to show degree assortativity^38^ and the RG graphs in our dataset are highly assortative with assortativity $\sim 0.55$. However, ER graphs have degree assortativity close to 0. The similarity between the DD distributions in RG and ER graphs reveals a remarkable fact about these two synthetic networks; while they differ significantly in GA, the mixing patterns are strikingly similar at the local scale.

**Figure 7:**
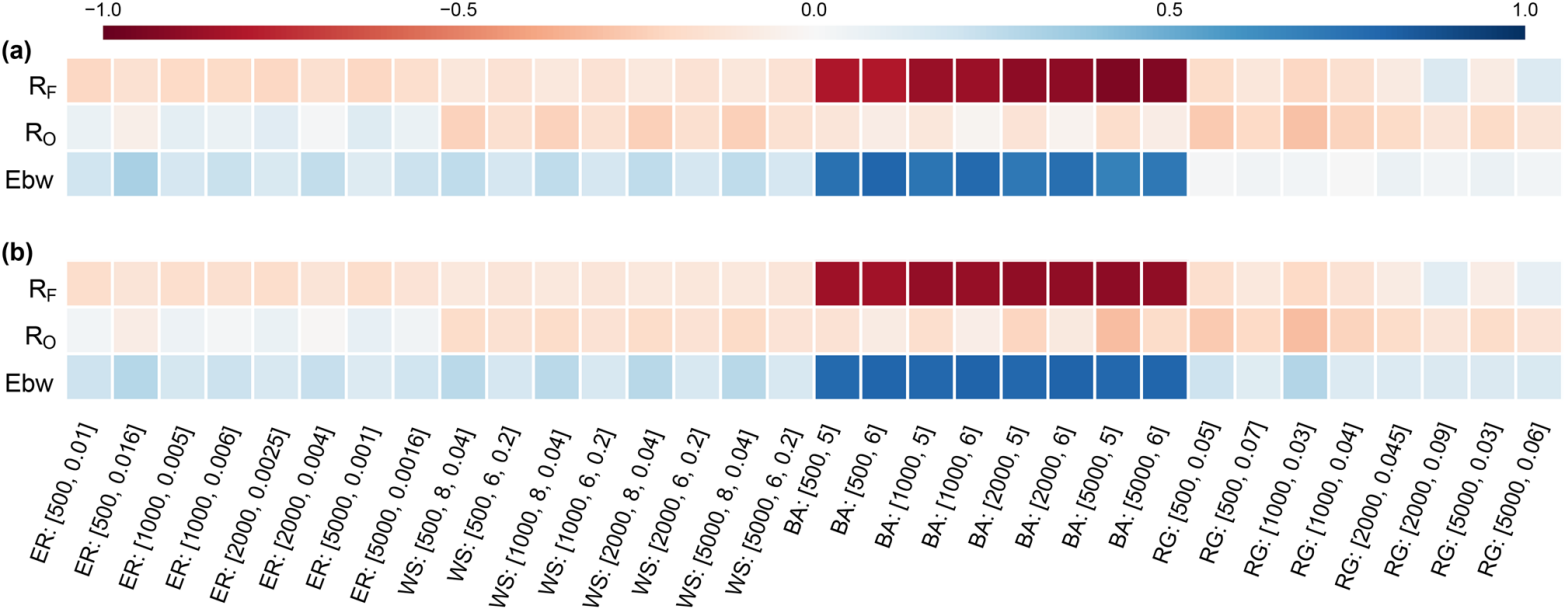
Correlation between degree difference (DD) and three established edge-based measures, namely, Forman–Ricci curvature ($R_F$), Ollivier–Ricci curvature ($R_O$), and edge betweenness centrality (Ebw) in synthetic networks. We show in (**a**) the Pearson correlation coefficients and in (**b**) the Spearman correlation coefficients. For each model, the parameters used are indicated besides it in parenthesis. For ER model, the parameters are number of vertices *n* and probability *p* of connecting an edge between any pair of vertices. For WS model, the parameters are number of vertices *n*, the number of neighbours *k* to which each vertex is connected in the starting regular graph, and rewiring probability $\beta$. For BA model, the parameters are number of vertices *n* and number of edges $\beta$ that are attached to the new vertex at each iteration step. For RG model, the parameters are number of vertices *n* and radius $\epsilon$. The reported correlation for each model and a given set of parameters is an average over a sample of 50 networks.

### DD distribution in directed networks

DD distribution can be computed in directed networks by considering the networks as undirected by ignoring the directions on edges. Such a computation of DD distribution in undirected simplifications of directed networks could still be informative of existing heterogeneity as demonstrated for the 4 directed real networks, namely, Citation, Email, Metabolic, and WWW, in Fig. 6. To better understand the details of this heterogeneity though, we can use a directed variation of DD that can highlight the specifics leading to such heterogeneity.

The directed DD (diDD) as defined in Eq. (6), captures the local homophily between in-degree of the tail vertex and out-degree of the head vertex of a directed edge. One can also define diDD differently in order to study homophily between other combinations of in-degree and out-degree of vertices anchoring a directed edge. In fact, distributions of diDD defined with respect to each combination of in-degree and out-degree can be informative in their own right. For instance, we consider diDD, $\daleth _{\rightarrow {}}$ as defined in Eq. (6) as well as an alternate variation of diDD defined as $\daleth ^{alt}_{\rightarrow {}} := {\text {deg}}^{out}({\text {u}}) - {\text {deg}}^{out}({\text {v}})$ for a directed edge (*v*, *u*). The diDD distributions in Fig. 6 enable us to make the following observations about the 4 directed real networks in our dataset. Metabolic network has a rather symmetric homophily in the direction of the reactions, for both variations of diDD. On the other hand, for Citation network, while distribution of $\daleth ^{alt}_{\rightarrow {}}$ is relatively symmetric for the negative and positive values, the distribution of $\daleth _{\rightarrow {}}$ is rather asymmetric with a long tail in the negative side. In Email network, the majority of Emails are exchanged between Email addresses with similar Email traffic. In the distributions for Email network, there are two other major peaks corresponding to Emails sent from Email addresses sending many Emails (e.g. organizational Email addresses) to those that send only few Emails (Fig. 6j). There are also a small number of Emails sent from Email addresses receiving only a small number of Emails to those sending out a large number of Emails (Fig. 6f). Moreover, according to the distributions in WWW network, within the domain of University of Notre Dame, there are many hyperlinks from webpages that have links to many other webpages (e.g. a departmental webpage) to those that do not contain many hyperlinks to other webpages (e.g. a webpage corresponding to an announcement) (Fig. 6l).

**Figure 8:**
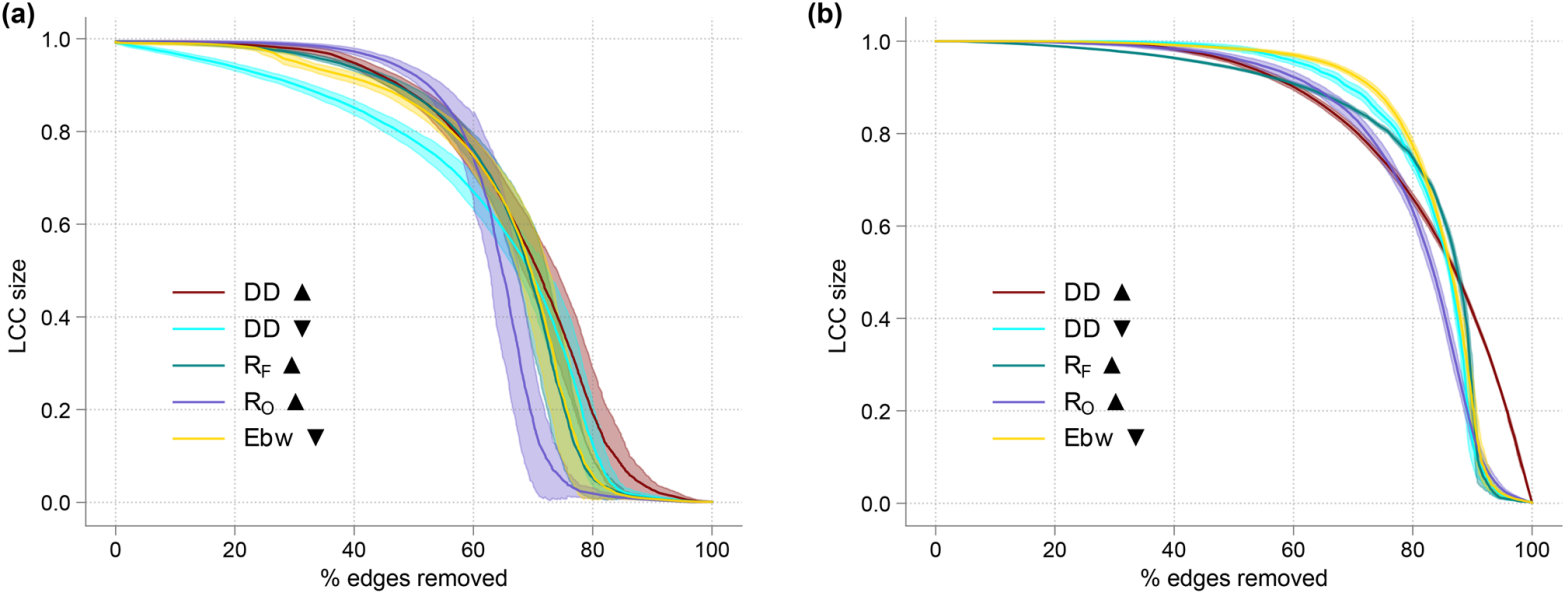
Topological robustness of synthetic networks with respect to deletion of edges based on DD, Forman–Ricci curvature ($R_F$), Ollivier–Ricci curvature ($R_O$), and edge betweenness centrality (Ebw). (**a**) Erdös–Rényi (ER) network with $n=1000$ and $p=0.01$. (**b**) Barabási–Albert (BA) network with $n=1000$ and $\beta =5$. In each case, we show the size of the largest connected component (LCC) normalized by the number of vertices in the graph as a function of the percentage of edges removed. Edges are deleted based on increasing and decreasing order of DD, increasing order of $R_F$, increasing order of $R_O$ and decreasing order of Ebw. For each model, the plots show the mean and standard deviation of LCC size over an ensemble of 50 networks generated with the specified parameters.

### Correlation with other edge-based measures

We explore the correlation between DD and three other established edge-based measures, namely edge betweenness centrality^39,40^, Forman-Ricci curvature ($R_F$)^13,14^ and Ollivier-Ricci curvature ($R_O$)^11,12,14,16^ for characterizing the local network geometry. These results are summarized in Fig. 7. It is seen that DD is moderately correlated with edge betweenness and $R_F$ in BA networks, and this correlation is positive with edge betweenness and negative with $R_F$. To avoid misinterpretation, however, it is important to note that the degree sum enters negatively into the definition of $R_F$ in the case of unweighted and undirected graphs, due to the fact that this notion originated in Riemannian geometry and therefore carries over the normalizations natural in that field. In SI Figure S3, we show the distribution of $R_F$ in the 4 classes of synthetic networks analyzed here. By comparing with Fig. 4, it is seen that DD provides insight into the structural heterogeneity of a network, which is not captured by Forman–Ricci curvature. In essence, we find that degree sum and degree difference are positively correlated in scale-free BA networks, which seems of interest for further understanding of those networks. In general, however, one does not expect such a correlation, and indeed, the correlation of DD with $R_O$ in all 4 classes of synthetic networks analyzed here, and with $R_F$ in all classes other than BA networks, seems to be negligible. Although edge betweenness seems to have a weakly positive correlation with DD across four classes of synthetic networks, this correlation seems to be noticeable only for BA networks while being sufficiently small in RG graphs.

These observations further clarify that DD distribution, despite its connection with measures such as edge betweenness and discrete Ricci curvatures, is an independent measure. As explained in other subsections, DD distribution as a stand-alone measure can be informative for the local geometry of the edges in the network and heterogeneity in mixing patterns, and other edge-based measures considered here cannot be used as a canonical proxy for DD.

### DD distribution and topological robustness

To test any potential relationship between DD value of edges and topological robustness of the network, we here compute the expected size of the largest connected component (LCC) in two ensembles of ER and BA networks during reverse edge percolation in increasing and decreasing order of DD. Through a comparative analysis, we also investigate the importance of DD for finding the minimum edge cut of the LCC.

Figure 8 shows the result of this reverse edge percolation analysis in ER and BA networks with respect to increasing and decreasing order of DD, increasing order of Forman–Ricci curvature ($R_F$), increasing order of Ollivier–Ricci curvature ($R_O$), and decreasing order of edge betweenness. In case of the BA network, this specific simulation shows a second-order phase transition when edges are removed in decreasing order of DD, a phenomenon observed for edge removal in increasing order of $R_F$. Moreover, in BA networks, the impact of failure of edges in decreasing order of DD on LCC size seems to be only negligibly different from when failure happens in decreasing order of edge betweenness. This similarity in BA networks is not simply due to the moderate positive correlation between these two measures, but has to do with the importance of local geometry for global connectivity in these networks. In other words, removing edges with large DD seems to be as detrimental to the LCC size as is removing edges with large edge betweenness, although the former, in contrast to the latter, depends only on the local geometry of the network. Thus, for purposes of robustness, the easily-computable and local DD can be a good proxy for the global edge betweenness. Therefore, edges with large DD play important roles for the global coherence in a network and they deserve systematic attention.

**Figure 9:**
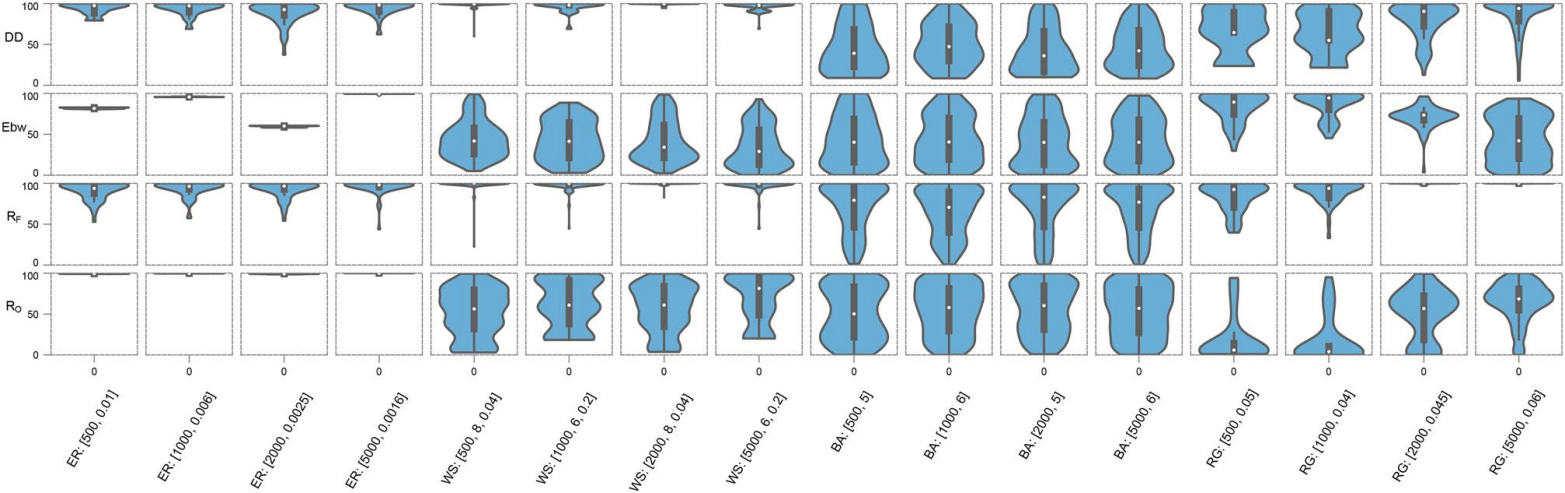
Comparison of the importance of the four edge-based measures, DD, edge betweenness (Ebw), Forman–Ricci curvature ($R_F$), and Ollivier–Ricci curvature ($R_O$), towards predicting the minimum edge cut (MEC) in synthetic networks. For the MEC of the LCC in each synthetic network, we determine the percentile of each edge in the MEC with respect to the value of the four measures on the edges. Then, we pool the edge percentiles for MEC corresponding to each network in the ensemble. Each violin plot shows the distribution of these edge percentiles in the pool corresponding to 50 networks in the ensemble for the corresponding synthetic network. For each model, the parameters used are indicated besides it in parenthesis as described in caption of Fig. 4.

The minimum cut^41^ in a connected network is another factor that is an indicator of topological robustness. Minimum edge cut (MEC) is a set of edges of minimum size that, if removed, the initially connected network is no longer one connected component. In Fig. 9, we compare the importance of each of the four edge-based measures—DD, edge betweenness, $R_O$ and $R_F$—towards predicting the MEC in the network. We compute the MEC of the LCC in each of the 4 synthetic networks analyzed here, and then, determine the percentile of each edge in the MEC with respect to the value of the measure on the edges in the LCC. Thereafter, we pool the percentiles of the edges for MEC corresponding to each network in the ensemble. In Fig. 9, each violin plot shows the distribution of these edge percentiles in the pool corresponding to 50 networks in the ensemble for each synthetic network. This figure shows that MEC in BA networks seems to be rather uncorrelated with these edge-based measures. In RG graphs, while only edge betweenness and the two curvature measures show some potential for being used to infer MEC in sparser RG graphs, for denser RG graphs, where most edges in the MEC seem to be almost flat with respect to Forman-Ricci curvature, DD appears as the second best predictor of MEC. These two best predictors of MEC in dense RG graphs are, however, the worst predictors of MEC in ER networks. Notably, DD seems to be the most important measure for inferring MEC in Watts–Strogatz (WS) small-world networks, especially when the network is highly regular. Thus, this simple measure seems to play an important role in keeping the LCC connected, or in other words, having a larger LCC, in a variety of network structures.

## Conclusions

Unravelling the structure of complex networks is a key interest since the rise of network science. To better understand the structure of large networks, it is necessary to study both the global macro-scale properties and the local features from which the global network structure emerges. Heterogeneity and homogeneity in mixing patterns of vertices in complex networks is an important known characterizing feature of network structure, which reveals features beyond the degree sequence. Degree assortativity was famously introduced to quantify such heterogeneity at a global scale. In this contribution, we study degree difference (DD) as the basic unit of mixing pattern in complex networks and explain the significance of this local edge-based measure. We explain how this simple, elegant, and computationally inexpensive measure can reveal valuable information about network structure. A closely related measure, namely degree-degree distance, which is defined as the difference of the logarithm of the degrees of both vertices constituting an edge, has been recently introduced and used to study scale-free property in complex networks^21^. In this work, we systematically explored DD as a measure for structural analysis of complex networks and discuss the mathematical connection between DD and global assortativity. Note that degree-degree distance^21^, unlike DD studied here, is less closely related to assortativity. Furthermore, we show that DD can be used to characterize the local network geometry and shed light on an understudied source of similarities or differences between different classes of synthetic and real networks. Notably, our numerical and analytical computations speak to independence and usefulness of this measure in its own right, as well as its importance for topological robustness of networks. In conclusion, we recommend the simple measure, degree difference, to be included in the standard toolkit of network science.

Moving forward, we expect this research will seed additional studies on local structural properties of complex networks in both theoretical and applied settings. As the transition from local to global mixing patterns in complex networks is yet to be systematically explored, we believe further theoretical and empirical studies on heterogeneity in mixing patterns at various scales of coarse-graining can help improve our understanding of the mesoscale network structure and how the global topology of complex networks emerges from its local geometry.

## Supplementary information

Supplementary Information.

## Data Availability

All data generated or analyzed during this study are included in this article or is available upon request from the corresponding author.
