# Supplementary material for: Degree difference: a simple measure to characterize structural heterogeneity in complex networks
Source: Sci Rep. 2020 Dec 7;10:21348. doi: 10.1038/s41598-020-78336-9 (PMC7721722; doi:10.1038/s41598-020-78336-9)
Supplement: Supplementary file 1 — Supplementary Information. [file 41598_2020_78336_MOESM1_ESM.pdf]

Supplementary Information
for
Degree difference: A simple measure to characterize structural heterogeneity in complex networks

Amirhossein Farzam[1,*], **Areejit Samal**[1,2,*], **and Jürgen Jost**[1,3,*]

[1]Max Planck Institute for Mathematics in the Sciences, Leipzig 04103 Germany
[2]The Institute of Mathematical Sciences (IMSc), Homi Bhabha National Institute (HBNI), Chennai 600113 India
[3]The Santa Fe Institute, Santa Fe, New Mexico 87501 USA
[*]Correspondence: farzam@mis.mpg.de; asamal@imsc.res.in; jost@mis.mpg.de

Appendix

In this paper, we studied the DD distribution in a number of synthetic and real-world networks. Moreover, we have used this dataset for an empirical analysis of the properties of DD measure, its significance in revealing topological features of networks, and its correlation with other network measures.
The synthetic networks considered in our analysis are as follows:

- **Erdös-Rényi (ER) random graphs**[1] **:** This model generates network with n vertices and between any given pair of vertices, there exists an edge with probability p.
- **Watts-Strogatz (WS) small-world graphs**[2] **:** This model generates network by starting with a k-regular (ring) lattice with n vertices, and then, each edge is randomly rewired with probability β. The model gives networks with small-world property, i.e., with small average path length and high clustering coefficient.
- **Barabási-Albert (BA) scale-free graphs**[3] **:** This model generates network via a preferential attachment scheme, wherein at each step, β new edges connect a new vertex to existing vertices v with probability proportional to $deg(v)$. These networks display power-law degree distribution and scale-free property.
- **Random geometric (RG) graphs**[4] **:** This model generates network with n vertices, each taking a random position in a 2-dimensional Euclidean plane. Thereafter, by fixing a radius parameter ε for the network, each vertex v is connected to all other vertices that fall inside the ball $B_\varepsilon(v)$ centered at v.

We have also analyzed the following undirected real-world networks:

- **Actor**[5]**:** This is a co-stardom network with 702388 actors as its vertices and 29397908 edges connecting those actors who appeared in at least one movie together.
- **Collaboration**[6]**:** Condensed Matter Physics collaboration network with 23133 vertices corresponding to authors who authored papers posted on arXiv during the period from January 1993 to April 2003. This network has 93439 edges with each edge between two vertices (authors) signifying co-authorship in at least one paper.
- **Internet**[7]**:** This is network of 192244 routers (vertices) with 609066 connections (edges).
- **Phone calls**[8]**:** This network captures phone calls between a sample of active cell phone users. In this network, there are 36595 users represented as vertices with 56853 edges between them. Two vertices are connected with an undirected edge if the corresponding users have at least once made a phone call to each other over the observed time interval.
- **Power grid**[2]**:** This network represents the power grid in western states of USA. Vertices are power plants and edges represent direct connections between power plants via a cable. In this network, there are 4941 vertices and 6594 edges between them.
- **Protein**[9] **:** This network is a human protein-protein interaction network with 2018 proteins as vertices and 2930 edges which represent mutual engagement of a pair of proteins in an interaction.

In addition to the above-mentioned undirected real networks, we have also analyzed the following directed real networks:

- **Citation[10]:** This is a network of citations between 449673 papers (vertices) published in APS journals. A directed edge points from a vertex v to a vertex u if v cites u. There are 4685576 directed edges in this network.
- **Email[11]:** This network is based on Email communications at the University of Kiel, Germany over 112 days. There are 57194 vertices, which are the email addresses, and there is a directed link from vertex i to vertex j if i has sent at least one email to j. Overall, there are 93090 directed edges in this network.
- **Metabolic[12]:** This is a network of metabolic reactions in bacterium *E. coli* where vertices are metabolites and directed edges are reactions linking reactants to products of reactions. This network contains 1039 metabolites as vertices and 4741 reactions as directed edges.
- **WWW[13]:** This is a network of hyperlinks within nd.edu domain. In this network, vertices are webpages and there is a directed edge from a webpage v to a webpage u if v includes at least one hyperlink to u. This network contains 325729 vertices and 1117563 edges.

References

1. Erdös, P. & Rényi, A. On the evolution of random graphs. Bull. Inst. Internat. Stat. **38**, 343–347 (1961).
2. Watts, D. J. & Strogatz, S. H. Collective dynamics of small-world networks. Nature **393**, 440–442 (1998).
3. Barabási, A. L. & Albert, R. Emergence of scaling in random networks. Science **286**, 509–512 (1999).
4. Dall, J. & Christensen, M. Random geometric graphs. Phys. Rev. E **66**, 016121 (2002).
5. Barabási, A.-L. Network science (Cambridge University Press, 2016).
6. Leskovec, J., Kleinberg, J. & Faloutsos, C. Graph evolution: Densification and shrinking diameters. ACM transactions on Knowl. Discov. from Data (TKDD) **1**, 2 (2007).
7. Center for applied internet data analysis (caida). Retrieved from http://www.caida.org/tools/measurement/skitter/router_topology/.
8. Song, C., Qu, Z., Blumm, N. & Barabási, A.-L. Limits of predictability in human mobility. Science **327**, 1018–1021 (2010).
9. Yu, H. et al. High-quality binary protein interaction map of the yeast interactome network. Science **322**, 104–110 (2008).
10. Redner, S. Citation statistics from more than a century of physical review. Preprint at https://arxiv.org/abs/physics/0407137 (2004).
11. Ebel, H., Mielsch, L.-I. & Bornholdt, S. Scale-free topology of e-mail networks. Phys. Rev. E **66**, 035103 (2002).
12. Schellenberger, J., Park, J. O., Conrad, T. M. & Palsson, B. Ø. BiGG: a biochemical genetic and genomic knowledgebase of large scale metabolic reconstructions. BMC Bioinforma. **11**, 213 (2010).
13. Albert, R., Jeong, H. & Barabási, A.-L. Diameter of the world-wide web. Nature **401**, 130–131 (1999).

Supplementary Figures

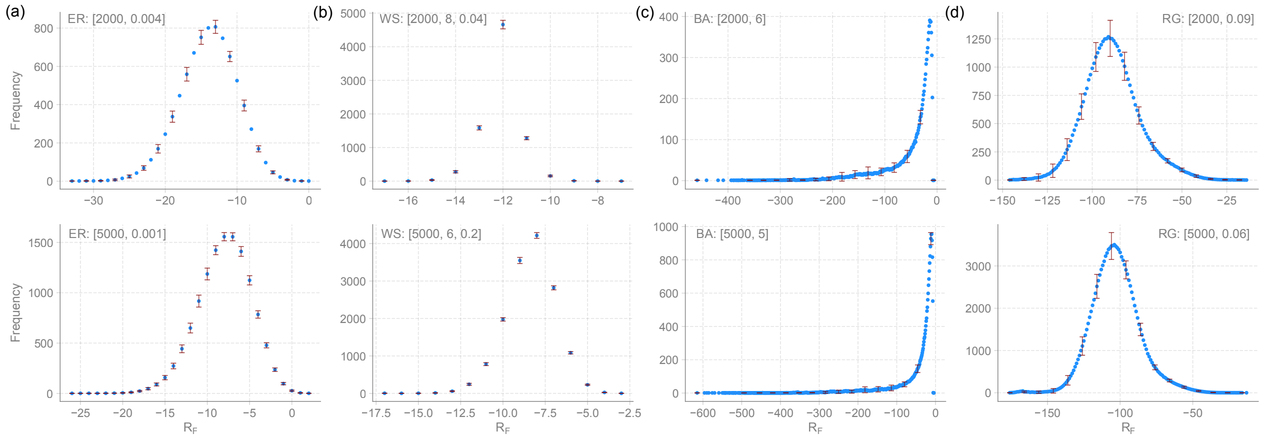

Figure S1. Forman-Ricci curvature (R_F) distributions in 4 different synthetic networks. (a) Erdös-Rényi (ER). (b) Watts-Strogatz (WS). (c) Barabási-Albert (BA). (d) Random Geometric (RG). For each network model, the parameters used are indicated besides it in parenthesis. For ER model, the parameters are number of vertices n and probability p of connecting an edge between any pair of vertices. For WS model, the parameters are number of vertices n, the number of neighbours k to which each vertex is connected in the starting regular graph and rewiring probability β. For BA model, the parameters are number of vertices n and number of edges β that are attached to the new vertex at each iteration step. For RG model, the parameters are number of vertices n and radius ε. The reported distribution for each network model and a given set of parameters is an average over a sample of 50 networks, shown as dots, and the error bars show the corresponding standard deviation.

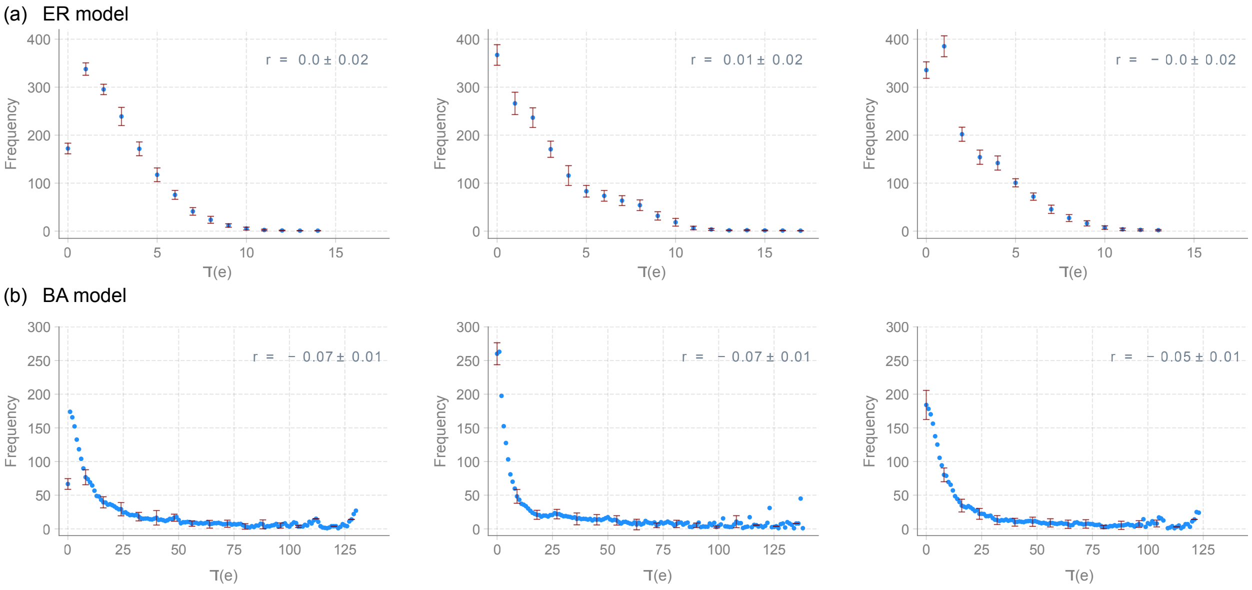

Figure S2. DD distributions for a given synthetic network and two rewired networks with same degree sequence as the given network and with pairwise global assortativity difference of ≤ 0.025 with respect to the given (starting) network. (a) Erdös-Rényi (ER) networks with $n = 500$ and $p = 0.012$. (b) Barabási-Albert (BA) networks with $n = 500$ and $\beta = 5$. In each subfigure, we show the average and standard deviation of the DD values over an ensemble of 20 networks as dots and error bars, respectively. In each plot, the legend gives the average and standard deviation of the global assortativities for the ensemble of 20 networks. Interestingly, although the global assortativity of each rewired network is ≤ 0.025 different from the starting network, and the difference between global assortativities of the two rewirings is ≤ 0.05, difference in DD distribution is clearly visible.

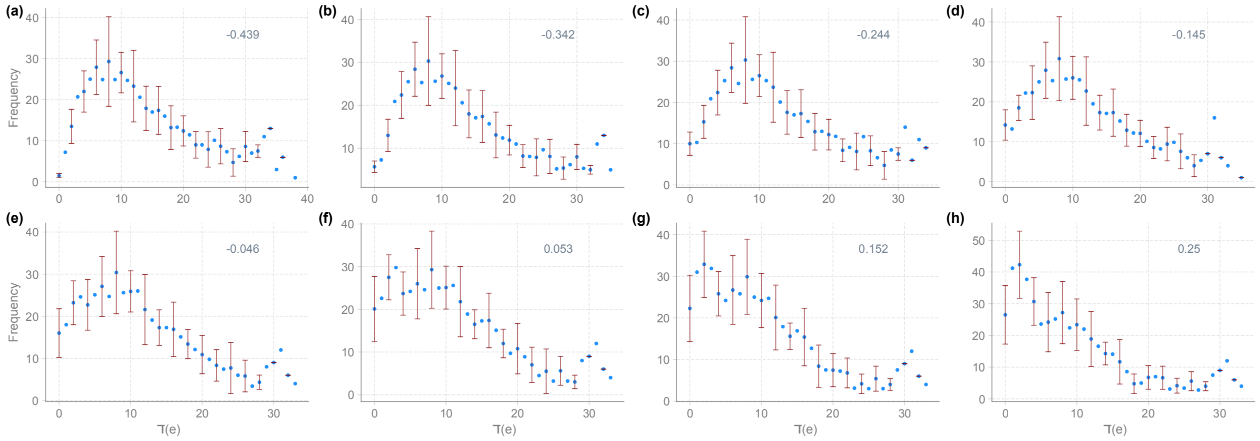

Figure S3. The evolution of DD distribution of Barabási-Albert (BA) networks created with $n = 100$ and $\beta = 5$ wherein the degree sequence is kept fixed while global assortativity differs. We start with a disassortative network and gradually increase the assortativity through a targeted rewiring scheme. Briefly, this heuristic to increase the assortativity of a given network is as follows. Given the graph at time step t, $G_t(V,E)$, we randomly pick two edges, $\{v,u\}$ and $\{w,z\}$ from E. We then remove the edges out of the network to obtain $\hat{G}(\hat{V},\hat{E})$, and relabel v, u, w and z to v_1, v_2, v_3, and v_4 where the vertices are indexed in the decreasing order of their degree. We next add a pair of edges $\{v_1,v_2\}$ and $\{v_3,v_4\}$ to $\hat{E}$. If the assortativity of $\hat{G}(\hat{V},\hat{E})$ is greater than that of G_t, we accept the change, and initialize G_{t+1} to $\hat{G}(\hat{V},\hat{E})$, otherwise, we discard the change and initialize G_{t+1} to G_t. We continue this process for a fixed number of time steps to obtain a more assortative network in comparison to the starting network. In this figure, we show the evolution of DD distributions for an ensemble of 20 BA networks with $n = 100$ and $\beta = 5$ starting as disassortative networks in subfigure (a) and evolving to assortative networks in (h). In each subfigure, we show the average and standard deviation of the DD values over an ensemble of 20 networks as dots and error bars, respectively. The ensemble average of the assortativity values are specified in the legend of each plot from (a)-(h).
